# Research and Innovation Opportunities to Improve Epidemiological Knowledge and Control of Environmentally Driven Zoonoses

**DOI:** 10.5334/aogh.3770

**Published:** 2022-10-21

**Authors:** Tatiana Proboste, Ameh James, Adam Charette-Castonguay, Shovon Chakma, Javier Cortes-Ramirez, Erica Donner, Peter Sly, Ricardo J. Soares Magalhães

**Affiliations:** 1 UQ Spatial Epidemiology Laboratory, School of Veterinary Science, University of Queensland, Gatton, Australia; 2 Queensland Alliance for One Health Sciences, School of Veterinary Science, University of Queensland, Gatton, Australia; 3 Children’s Health and Environment Program, Child Health Research Centre, The University of Queensland, Brisbane, 4101 QLD, Australia; 4 Centre for Data Science, Queensland University of Technology, Kelvin Grove, 4059 QLD, Australia; 5 Future Industries Institute, University of South Australia, Mawson Lakes, SA 5095, Australia; 6 Children’s Health and Research Centre, Children’s Health and Environment Program, The University of Queensland, South Brisbane, Australia

**Keywords:** Environmentally driven zoonoses, One Health, Zoonoses, Antimicrobial resistant, Animal health

## Abstract

While zoonotic diseases are defined by transmission processes between animals and humans, for many of these diseases the presence of a contaminated environmental source is the cause of transmission. Most zoonoses depend on complex environmentally driven interactions between humans and animals, which occur along an occupational and recreational environmental continuum, including farming and animal marketing systems, environmental management systems, and community leisure environments.

Environmentally driven zoonoses (EDZs) are particularly challenging to diagnose and control as their reservoirs are in the natural environment and thus often escape conventional surveillance systems that rely on host monitoring. Changes in the environment as a result of climate change [1], human population density [2], and intensification of agriculture [3] have been linked to increasing transmission events for this group of infections. As such, there is a recognised need to be able to detect the presence of EDZs in the environment as a means to better anticipate transmission events and improve source attribution investigations. Finally, the recognition that a One Health approach is needed to combat these infections is signalling to governments the need to develop policy that optimises trade-offs across human, animal, and environmental health sectors.

In this review, we discuss and critically appraise the main challenges relating to the epidemiology, diagnosis, and control of environmental zoonotic disease. Using a set of exemplar diseases, including avian influenza and antimicrobial resistant pathogens, we explore the epidemiological contexts (risk factors) within which these infections not only impact human health but also contribute to animal health and environmental impacts. We then critically appraise the surveillance challenges of monitoring these infections in the environment and examine the policy trade-offs for a more integrated approach to mitigating their impacts.

## Introduction

**Figure 1:**
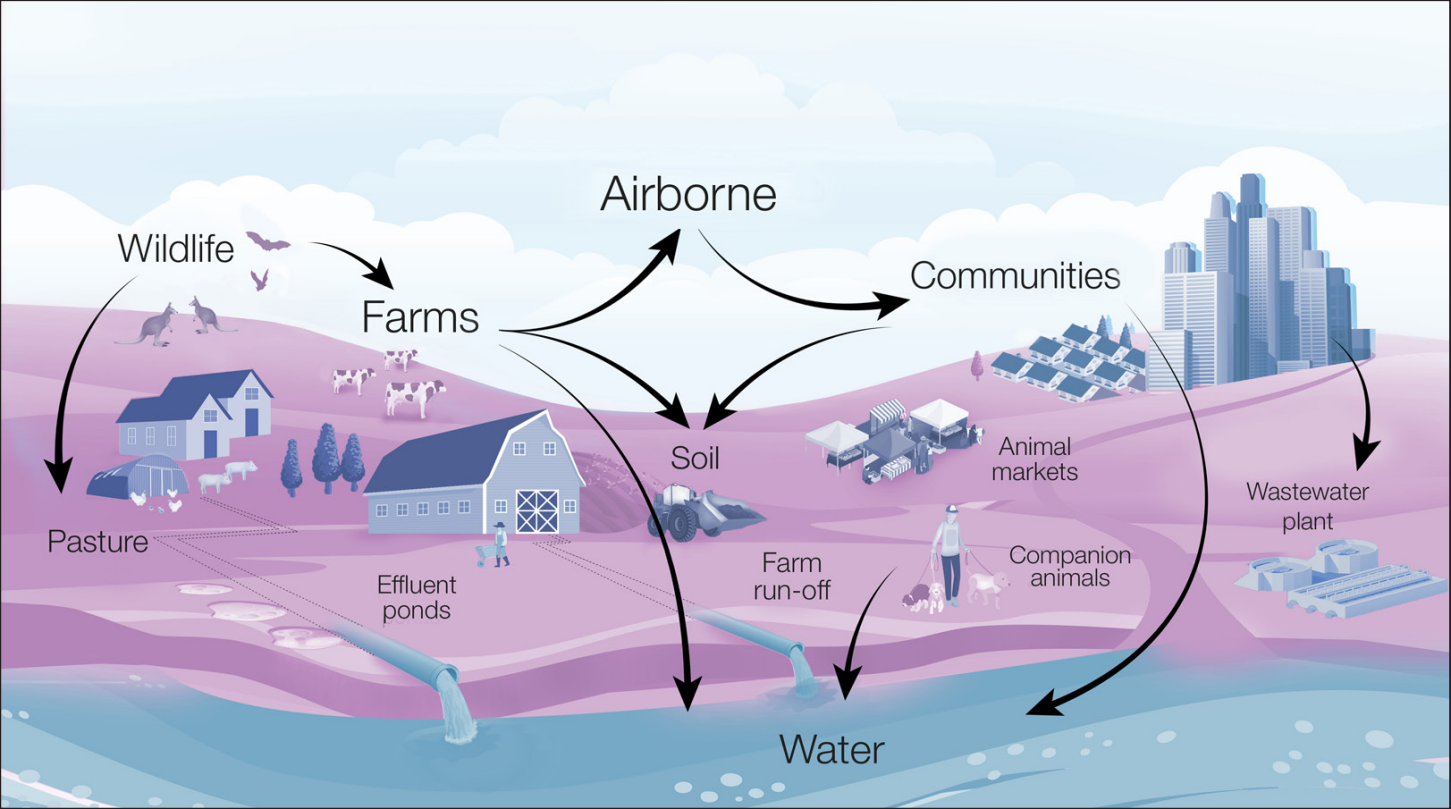
Potential routes of transmission of environmentally driven zoonoses along the landscape gradient of human-animal-environment interactions.

The transmission mechanisms of zoonotic pathogens that rely on the environment to be passed on to human or animal hosts (i.e., livestock, companion animals and wildlife) are complex and diverse (Figure 1). Infected animals can shed pathogens through bodily fluids, afterbirth products, faeces, and milk. These modes of pathogen excretion result in the contamination of the surrounding environment such as soil, air, and water bodies, which may then act as form reservoirs of infectious agents that subsequently transmit to other animals and people interacting with these environments. Cholera, for instance, is transmitted through the ingestion of water contaminated with *Vibrio cholerae*, and is closely linked to poor sanitation and hygiene. Q fever is another example, but this is primarily transmitted by the inhalation of dust particles contaminated with *Coxiella burnetii* and is typically associated with animal industries.

The epidemiology of environmental-driven zoonotic (EDZ) and the capability of pathogens to spread in the environment are affected by multiple factors, including changes in the natural environment. The growing world population has resulted in a higher demand for food and expansion of agricultural land. The encroachment of farmland into wildlife habitats is an important ecological driver of environmentally driven zoonoses as it enhances the probability of livestock exposure to wildlife reservoirs of infection. The proximity between livestock and wildlife species promotes zoonotic pathogen transmission through the contamination of livestock feed and water sources by wildlife excreta harbouring important zoonotic pathogens. For example, wildlife habitat encroachment was found to lead to higher risks of SARS-related coronavirus outbreaks [4]. The impact of future encroachment of peri-urban settlements on cropland, and the impact of climate variation on biodiversity hotspots and future transmission of EDZs to human and livestock populations, will require further modelling work to enable identification hotspots of human and livestock exposure to environmental and wildlife reservoirs of infection.

The environmental success of soil-transmitted, airborne, and waterborne infections is determined by the survival of the pathogen in these substrates. For example, the bacteria *Coxiella burnetii*, causative agent of Q fever in humans and coxielosis in ruminants, is categorised as an airborne pathogen. *Coxiella burnetii* can survive up to 10 months at 15–20°C and travel by wind (in contaminated dust particles) up to ~20 km from the primary source [5]. Furthermore, the transmission efficiency of an environmentally driven pathogen is determined by its ability to be transmitted via multiple pathways of infection increasing the difficulty understanding their epidemiology. Using the example of *C. burnetii*, the main pathway of human and animal infection is through the inhalation of contaminated aerosols with extracellular forms of *C. burnetii* [5,6,7,8], but drinking contaminated raw milk has also been described as a potential pathway of infection [9].

These human-livestock-wildlife interactions are expected to intensify as the world’s urban areas are projected to increase between 21 and 72% by 2050 [10]. A study found that approximately 4% of the world’s biodiversity hotspots (900,000 km^2^) are expected to be converted to cropland by 2050, particularly in the Indo-Burma Hotspot [11]. For instance, previous work estimated that 50–63% of the newly expanded urban land over the next 30 years is expected to encroach on current croplands [12]. Some of these croplands may constitute environmental reservoirs and their conversion to urban or peri-urban presents significant risks of infection from highly-resistant diseases in abandoned agricultural areas, such as *C. burnetii*. These forecasted scenarios pose a number of policy conundrums for EDZs in that human-dominated landscapes, such as urban areas and croplands, are believed to have a particularly strong influence on disease patterns in wildlife, domestic animals, and human populations [13]. Anthropogenic activities resulting in degraded wildlife habitat quality have increased opportunities for animal–human interactions and facilitated zoonotic disease transmission [14]. Research into decision-science approaches to better EDZ policy will be needed to evaluate trade-offs between health risks, food security, livelihoods and environmental conservation associated with land use change, and inform policies and land use management that can minimise these trade-offs.

In this review we discuss novel modelling approaches to uncover the environmental determinants that influence EDZ distribution and transmission, critically appraise the opportunities for environmental surveillance approaches for monitoring them, and examine the policy trade-offs that must be considered when informing integrated approaches to mitigating the impacts of these infections.

## Systems-Based Modelling of the Determinants of Environmentally Driven Zoonoses

Measuring the contribution of environmental and sociodemographic factors on the incidence of EDZs is critical to understanding the risk of infectious disease. Yet this is a very challenging task [15]. EDZs appear in human populations as a result of a complex cascade of transmission events, often originating at the wildlife-livestock interface. Due to urban development, expansion of cities, and population growth, communities are now more likely to have contact with wildlife, either directly or indirectly, via contact with livestock or farmed environments. The expansion of urban areas and croplands tends to negatively impact biodiversity and provides suitable conditions for smaller animals, adaptable to human pressures, which are more likely to carry zoonotic diseases [16]. Furthermore, human population density and the proximity to species’ range have been found to be positively related to pathogen richness in mammals, leading to zoonotic “spillover” events [17]. The proximity of urban or peri-urban settlements to wildlife reservoirs is a major driver of several zoonotic infections. For instance, outbreaks of human Nipah virus encephalitis cases in Bangladesh and India are believed to have originated first from bats via contaminated date palm sap, and subsequently through human-to-human transmission [18].

To capture the complex causal pathway of EDZ infection risk into a harmonised disease modelling framework, there is a need to fully consider the environmental transmission pathways as well as approaches to model validation and calibration [19]. For example, a study in Queensland, Australia, used a Bayesian spatial hierarchical model to estimate the risk of hospitalisation due to EDZs [15]. The study found that an increase in average rainfall and occupational exposures were associated with an increased risk of hospitalisation for zoonotic diseases. While the identified changes in the morbidity patterns due to zoonotic diseases in Queensland can be partly attributed to spatial variations in environmental and occupational risk factors, there is a need to uncover the prognostic ability of clinical, sociodemographic, and contextual environmental characteristics of zoonotic disease hospitalised patients.

Most epidemiological studies of zoonoses use regression analyses that model theoretical representations of disease transmission and can restrict the consideration of other potential determinants such as multiple correlated features or traits across species [20]. Although traits can contribute to determine the development of zoonotic diseases (e.g., high population density and short lifespan can determine the infection period), these are usually not incorporated in conventional epidemiological studies. Since traits evolve from selection pressures in complex environments, functional traits can be used as proxies of characteristics that can determine host responses to pathogenic agents but are difficult to identify, such as immune strategies [21]. Machine learning methods have been increasingly used to assess the associations of multiple variables in big data sets where complex interactions are difficult to measure. This has been demonstrated in analyses using machine learning algorithms to study zoonotic vector status of mosquitoes and ticks and the zoonotic reservoir status of hosts [22,23,24]. Other methodological approaches, such as system dynamics, can be used in conjunction with machine learning to predict disease dynamics and potential spillovers by considering the characteristics of hosts in a given ecosystem [20].

With the global concern for the possible zoonotic origin of COVID-19, machine learning models have been particularly useful to predict the zoonotic capacity of vertebrate species to transmit the virus. Booster regression models that apply the gradient boosting machine learning algorithm have been collated with structural modelling that quantifies the virus protein receptor binding to the angiotensin-converting enzyme 2 receptor (ACE2) to predict potential spillback COVID-19 infections [25]. Since the broad host range of SARS-CoV-2 is linked to the ubiquity of ACE2 and the high prevalence of SARS-CoV-2 in humans, repeated spillback infections (i.e., human hosts infecting animals with SARS-CoV-2 virus) can determine the establishment of new animal hosts from which secondary spillover can lead to human infections [26,27]. Whereas structural models can predict how ACE2 homologous gene sequences across species bind to the viral spike protein, the prediction of host range for SARS-CoV-2 is restricted by the availability of ACE2 sequences between species. Combining structural modelling of viral binding with machine learning of species traits has increased the capacity to predict the zoonotic potential of SARS-CoV-2 across 5400 mammal species [25]. This combined approach can allow prediction for species for which ACE2 sequences are not available because it leverages data from the virus binding dynamics and biological traits.

## Integrated Approaches to the Environmental Detection of Environmentally Driven Zoonoses

The interconnectedness of EDZ transmission is facilitated by shared environments where humans, livestock, and wildlife interact. The environmental context of transmission such as soil, air, or water bodies act as a hub for many EDZ-causing pathogens. The quintessential example of an EDZ is the transmission of antimicrobial resistant pathogens. Antimicrobial resistance (AMR) is an increasing concern globally as it is estimated that 1.27 million death were directly caused by AMR [28] and about 10 million deaths are forecast in 2050. While the primary driver of AMR is the misuse of antibiotics in human and animal health settings and agriculture, there is increasing recognition of where and how AMR is emerging and being transmitted, especially from wastewater and soil, which ends up being accumulated in wastewater and soil [29,30]. AMR pathogens can be transmitted between humans, animals, and the environment through different routes, including ingestion and direct contact [31]. The environment has been identified as an important bridge for AMR transmission as it receives waste products from both humans and animal populations under selective pressure of antimicrobials and acts as a reservoir of clinically relevant AMR organisms, resistance genes, and antibiotic residues.

Wastewater (either influent or effluent) from communities (Figure 1) such as urban, hospital, and pharmaceutical plants have been reported as important reservoirs of clinically relevant AMR bacteria and resistance genes [29,32,33,34]. Both untreated and treated water from wastewater treatment plants can be environmental reservoirs of AMR organisms and resistance genes. AMR resistance genes are not degradable and therefore can contribute to the spread of AMR among microbial communities via horizontal gene transfer [35,36].

AMR has been surveyed and characterised with the help of molecular and phenotypic methods. These methods use polymerase chain reaction (PCR) coupled with Sanger sequencing to characterise AMR genotypes. Recently, whole genome sequencing (WGS) and metagenomic analysis have been deployed for extensive detection and characterization of AMR bacteria and resistance genes in wastewater samples. However, these approaches may not be suitable for immediate detection and decision-making in the field. Point of care tests (POCT) provide an option for quick and timely detection of AMR bacteria and resistance genes in wastewater.

There are emerging, emerged, and promising technologies that could be used in the field. Traditional phenotypic resistance tests occur in a microbiology laboratory, and this simply involves culture and antimicrobial susceptibility testing in 2-days. These tests are often subjective and user dependent and therefore are not amenable for field detection of AMR. Phenotypic tests that could detect phenotypic resistance to antimicrobials at POCT would be useful. Flexicult (SSI Diagnostica, Hillerød Denmark) is a user-friendly platform for pathogen culture and AMR detection at POCT, but overnight incubation of cultured plates is still required before results are interpreted; this may prolong decision-making and intervention at the field which will impact transmission of AMR from wastewater to humans. A more rapid phenotypic resistance test that could detect AMR from wastewater in 10–15 minutes would be highly regarded to mitigate transmission.

Rapid molecular technologies like isothermal amplification could provide the speed needed in the field. Among the isothermal technologies that have been extensively used in the development of rapid POCT is Loop mediated amplification (LAMP). LAMP has been used for the detection of *Listeria monocytogenes* from wastewater without DNA extraction in one hour using a water-bath set at 63∞C [37]. Also, this technology was recently used to detect SARS-CoV-2 (COVID-19) from wastewater [38]. Likewise, recombinase polymerase amplification (RPA) isothermal technology coupled with lateral flow strip has been shown to detect *Ascaris suum* ova in wastewater in less than 30 minutes at 37∞C [39]. Interestingly, in a proof of concept that used RPA coupled with microfluidic DNA chip, resistance gene (bla_CTX-M_) in water was detected and quantified in 40 minutes at 39∞C [40]. The isothermal diagnostic platform does provide an option for alternative detection of AMR organisms, but more research is required to demonstrate the potential of these technologies for detection of clinically relevant resistance genes from wastewater, which would help to improve control measures.

Finally, the increasing use of genome data can facilitate the incorporation of machine learning algorithms to identify the source of EDZs. For example, support vector machine algorithms have been used to study the relatedness of *E. coli* isolates in different hosts [41]. These analyses can be key for public health strategies in specific sectors as *E. coli* isolates from cattle have been found to have genetic information linked to isolates associated with infection in humans [42]. On the other hand, studies of human-like influenza virus in swine have implemented maximum likelihood analyses and random forest algorithms to identify gene sequences to differentiate human-origin from swine-origin A(H1N1) viruses [43]. The outcomes of these models can improve understanding of the biological host restrictions that these pathogens need to overcome to successfully infect a new host and indicate its potential zoonotic threat to humans.

Another important use of machine learning to predict potential zoonotic spillovers is its integration with networks of shared pathogens to assess the sharing and transmission of different pathogen taxa between mammals and mammalian reservoirs of zoonoses. Many studies that investigate distribution patterns of pathogens among mammals focus on determinants of pathogen sharing within some host groups (mostly primates, bats, carnivores and rodents) or are limited to certain pathogens, such as viruses [17,44,45]. However, machine learning algorithms allow a more comprehensive approach for all non-human mammalian hosts and humans by assessing the factors for various taxa of pathogens such as bacteria and viruses [46]. These integrative alternatives highlight the importance of multidisciplinary approaches including modelling, field surveillance, and laboratory experiments to quantify zoonotic risks and support prevention strategies [47]. The development of techniques to distinguish animal-origin pathogens that can be associated with human-origin strains is necessary for infectious diseases surveillance.

## Informing Interventions for Environmentally Driven Zoonoses Through the Identification of Multisectoral Synergies in Policy Formulation

Given the multidimensional nature and complex problem of EDZs, policies for control and prevention require a multi-pronged approach. Interventions critically need to identify synergies in policy interventions that support the improvement of animal and human health with the least economic and cultural impact on local communities. An example of opportunity for intervention measures occurs when we encounter a high density of animals (high contact rate), a high diversity of species (multi-host), and a confined environment, all conditions that are met during animal trade, such as in live bird markets (LBM). Despite the high risks to public health stemming from LBM, these markets provide multiple benefits to local communities. For instance, they greatly facilitate the accessibility to animal source foods, provide dietary diversity, and offer a cheaper option than supermarkets to low-income shoppers in several developing countries, hence contributing to food security in marginalised communities [48,49]. In addition, live animal markets can provide an additional source of income from tourism [50], can be more environmentally sustainable than industrial food systems [51], and have the potential to improve the resilience of the food supply chain by supporting different food-acquisition strategies of large vulnerable populations [52]. Live bird markets represent a key avenue for many smallholder producers, which produce the largest share of the food in developing countries, as opposed to products from large-scale, intensified, and often international farms sold in formal supermarkets [49]. Therefore, LBMs present important trade-offs between three major sustainable development goals, i.e., (1) reducing poverty, (2) achieving food security and improved nutrition, and (3) ensuring healthy lives [53].

Market closure and disinfection are effective ways to reduce the amount of viable virus [54,55]. However, because of the substantial socio-cultural benefits provided by live animal markets, it has been argued that, from a medium- and long-term perspective, negative impacts on nutrition and on the livelihoods of vendors associated with permanent closure of such markets may outweigh the health benefits [49,56]. Such intervention may have unintended consequences, for instance inducing changes in the movement and trade of contaminated animals that can result in the spread of microorganisms outside of the original market, e.g., to markets in other regions [57]. In addition, permanent closures risk a shift of activities to informal markets, where biosecurity measures may become more difficult or impossible to implement [58].

Other control measures can contribute to mitigating the risk of contamination before closures become necessary, to include reducing the size of markets, selling single poultry species, performing cleaning and disinfection, banning overnight storage, and sourcing poultry from local areas; other useful measures could include installing handwashing facilities and toilets, providing adequate drainage, separating different species of live animals from meat and produce, and implementing protocols for cleaning food and slaughtering animals [59,60].

Surveillance can be costly, and interventions should be prioritized where the risks are the highest. For instance, surveillance that prioritises market operations (e.g., wholesale, retail, both wholesale and retail), animal species, or seasons and time periods presenting higher risks of contamination can be a way to limit costs [61]. Previous studies have shown it is possible to optimize early detection of avian influenza in live bird markets by minimising trade-offs between surveillance costs and the number of infected birds in live markets at time of detection [62]. However, more research is needed to assess the capacity of predictive models to anticipate outbreaks based on environmental factors and decision support tools that can consider multiple criteria to identify risks and minimise trade-offs, e.g., the article by M.C. Paul et al [63].

Participatory disease surveillance and community-based reporting systems can significantly increase case detection in countries at risk of experiencing avian influenza and improve our understanding of the epidemiological situation as the local communities are the most vulnerable to socio-economic impacts of market closures [64]. Policy homogeneity is easier to design, politically appealing, and tends to be cheaper to implement in the short term. Fit-for-purpose surveillance systems that consider heterogeneity, localism, and consultation might be more expensive, but are likely to be more cost-effective in both the short and longer term [65].

Designing policies adapted to local stakeholders that can achieve One Health objectives and Sustainable Development Goals will require interdisciplinary research to analyse trade-offs and synergies between health, environmental, economic, and cultural interests, particularly given the increasing demand for animal source foods and population density in emerging countries.

## Concluding Remarks

Due to the complex interactions between their different hosts and the natural environment, EDZs pose a number of challenges with regard to the measurement of their determinants, the development of environmental detection methods, and the formulation of integrated policies for their control. One of the main gaps is the lack of understanding of the environmental and sociodemographic factors that affect the risk of exposure. Incorporating multiple correlated datasets, including environmental variables that are known to affect the survival, maintenance, and spread of EDZs into machine learning models can assist in obtaining better insights into these complex interactions and help predict areas of higher risk for better preparedness. Novel high throughput molecular techniques such as WGS and LAMP linked to machine learning algorithms have shown promise to better understand the pathogen-host-environment interaction for the development of detection and source attribution monitoring tools. The need for data-driven policy modelling approaches that fully consider the complexity in EDZ transmission is critical to inform disease control and prevention strategies [19]. Given the multidimensional nature of EDZs, to alleviate their burden in human and animal populations, effective and sustainable control and prevention measures must be informed by trade-offs between health, economic, and environmental objectives.

## References

[1] Rupasinghe R, Chomel BB, Martínez-López B. Climate change and zoonoses: A review of the current status, knowledge gaps, and future trends. Acta Tropica. 2022; 226: 106225. DOI: 10.1016/j.actatropica.2021.106225 34758355

[2] Allen T, et al. Global hotspots and correlates of emerging zoonotic diseases. Nature Communications. 2017; 8: 1–10. DOI: 10.1038/s41467-017-00923-8 PMC5654761 29066781

[3] Jones BA, et al. Zoonosis emergence linked to agricultural intensification and environmental change. Proceedings of the National Academy of Sciences of the United States of America. 2013; 110: 8399–8404. DOI: 10.1073/pnas.1208059110 23671097 PMC3666729

[4] Rulli MC, D’Odorico P, Galli N, Hayman DTS. Land-use change and the livestock revolution increase the risk of zoonotic coronavirus transmission from rhinolophid bats. Nat Food. 2021; 2: 409–416. DOI: 10.1038/s43016-021-00285-x 37118224

[5] Tissot-Dupont H, Amadei M-A, Nezri M, Raoult D. Wind in November, Q Fever in December. Emerging Infectious Diseases. 2004; 10: 1264–1269. DOI: 10.3201/eid1007.030724 15324547 PMC3323349

[6] Clark NJ, Soares Magalhães RJ. Airborne geographical dispersal of Q fever from livestock holdings to human communities: A systematic review and critical appraisal of evidence. BMC Infectious Diseases. 2018; 18: 1–9. DOI: 10.1186/s12879-018-3135-4 29764368 PMC5952368

[7] van der Hoek W, et al. Proximity to goat farms and *Coxiella burnetii* seroprevalence among pregnant women. Emerging Infectious Diseases; 2011. DOI: 10.3201/eid1712.110738 PMC3311170 22172140

[8] Tissot-Dupont HH, Torres S, Nezri M, Raoult D. Hyperendemic focus of Q fever related to sheep and wind. American Journal of Epidemiology. 1999; 150: 67–74. DOI: 10.1093/oxfordjournals.aje.a009920 10400556

[9] Gale P, Kelly L, Mearns R, Duggan J, Snary EL. Q fever through consumption of unpasteurised milk and milk products—a risk profile and exposure assessment. Journal of Applied Microbiology. 2015; 118: 1083–1095. DOI: 10.1111/jam.12778 25692216

[10] Gao J, O’Neill BC. Mapping global urban land for the 21st century with data-driven simulations and Shared Socioeconomic Pathways. Nature Communications. 2020; 11. DOI: 10.1038/s41467-020-15788-7 PMC7210308 32385275

[11] Molotoks A, et al. Global projections of future cropland expansion to 2050 and direct impacts on biodiversity and carbon storage. Global Change Biology. 2018; 24: 5895–5908. DOI: 10.1111/gcb.14459 30267559 PMC6282572

[12] Chen GZ, et al. Global projections of future urban land expansion under shared socioeconomic pathways. Nature Communications. 2020; 11. DOI: 10.1038/s41467-020-14386-x PMC6985221 31988288

[13] Hassell JM, Begon M, Ward MJ, Fevre EM. Urbanization and disease emergence: dynamics at the wildlife-livestock-human interface. Trends in Ecology & Evolution. 2017; 32: 55–67. DOI: 10.1016/j.tree.2016.09.012 28029378 PMC5214842

[14] Johnson CK, et al. Global shifts in mammalian population trends reveal key predictors of virus spillover risk. P Roy Soc B-Biol Sci. 287. DOI: 10.1098/rspb.2019.2736 PMC7209068 32259475

[15] Cortes-Ramirez J, Vilcins D, Jagals P, Soares Magalhaes RJ. Environmental and sociodemographic risk factors associated with environmentally transmitted zoonoses hospitalisations in Queensland, Australia. One Health. 2021; 12: 100206. DOI: 10.1016/j.onehlt.2020.100206 33553560 PMC7847943

[16] Gibb R, et al. Zoonotic host diversity increases in human-dominated ecosystems. Nature. 2020; 584: 398–+. DOI: 10.1038/s41586-020-2562-8 32759999

[17] Olival KJ, et al. Host and viral traits predict zoonotic spillover from mammals. Nature. 2017; 546: 646–650. DOI: 10.1038/nature22975 28636590 PMC5570460

[18] Rahman MA, et al. Date Palm Sap Linked to Nipah Virus Outbreak in Bangladesh, 2008. Vector-Borne and Zoonotic Diseases. 2012; 12: 65–72. DOI: 10.1089/vbz.2011.0656 21923274

[19] Rees EM, et al. Transmission modelling of environmentally persistent zoonotic diseases: a systematic review. The Lancet Planetary Health. 2021; 5: e466–e478. DOI: 10.1016/S2542-5196(21)00137-6 34245717

[20] Han BA, O’Regan SM, Schmidt JP, Drake JM. Integrating data mining and transmission theory in the ecology of infectious diseases. Ecology Letters. 2020; 23: 1178–1188. DOI: 10.1111/ele.13520 32441459 PMC7384120

[21] Lochmiller RL, Deerenberg C. Trade-offs in evolutionary immunology: just what is the cost of immunity? Oikos. 2000; 88: 87–98. DOI: 10.1034/j.1600-0706.2000.880110.x

[22] Han BA, et al. Confronting data sparsity to identify potential sources of Zika virus spillover infection among primates. Epidemics. 2019; 27: 59–65. DOI: 10.1016/j.epidem.2019.01.005 30902616

[23] Yang LH, Han BA. Data-driven predictions and novel hypotheses about zoonotic tick vectors from the genus Ixodes. BMC ecology. 2018; 18: 1–6. DOI: 10.1186/s12898-018-0163-2 29448923 PMC5815220

[24] Evans MV, Dallas TA, Han BA, Murdock CC, Drake JM. Data-driven identification of potential Zika virus vectors. elife. 2017; 6: e22053. DOI: 10.7554/eLife.22053 28244371 PMC5342824

[25] Fischhoff IR, Castellanos AA, Rodrigues J, Varsani A, Han BA. Predicting the zoonotic capacity of mammals to transmit SARS-CoV-2. Proc Biol Sci. 2021; 288: 20211651. DOI: 10.1098/rspb.2021.1651 34784766 PMC8596006

[26] World Health Organization. 2021.

[27] Chou CF, et al. ACE2 orthologues in non-mammalian vertebrates (Danio, Gallus, Fugu, Tetraodon and Xenopus). Gene. 2006; 377: 46–55. DOI: 10.1016/j.gene.2006.03.010 16781089 PMC7125734

[28] Murray CJL, et al. Global burden of bacterial antimicrobial resistance in 2019: a systematic analysis. Lancet. 2022; 399: 629–655. DOI: 10.1016/S0140-6736(21)02724-0 35065702 PMC8841637

[29] Fouz, N, et al. The contribution of wastewater to the transmission of antimicrobial resistance in the environment: implications of mass gathering settings. Tropical medicine and infectious disease. 2020; 5: 33. DOI: 10.3390/tropicalmed5010033 PMC7157536 32106595

[30] Armand-Lefevre L, Ruppé E, Andremont A. ESBL-producing Enterobacteriaceae in travellers: doctors beware. The Lancet Infectious Diseases. 2016; 17: 8–9. DOI: 10.1016/S1473-3099(16)30417-0 27751773

[31] Woolhouse M, Ward M, Van Bunnik B, Farrar J. Antimicrobial resistance in humans, livestock and the wider environment. Philosophical Transactions of the Royal Society B: Biological Sciences. 2015; 370: 20140083. DOI: 10.1098/rstb.2014.0083 PMC4424433 25918441

[32] Hocquet D, Muller A, Bertrand X. What happens in hospitals does not stay in hospitals: antibiotic-resistant bacteria in hospital wastewater systems. Journal of Hospital Infection. 2016; 93: 395–402. DOI: 10.1016/j.jhin.2016.01.010 26944903

[33] Bouki C, Venieri D, Diamadopoulos E. Detection and fate of antibiotic resistant bacteria in wastewater treatment plants: a review. Ecotoxicology and environmental safety 2013; 91: 1–9. DOI: 10.1016/j.ecoenv.2013.01.016 23414720

[34] McKinney CW, Dungan RS, Moore A, Leytem AB. Occurrence and abundance of antibiotic resistance genes in agricultural soil receiving dairy manure. FEMS Microbiology Ecology. 2018; 94: fiy010. DOI: 10.1093/femsec/fiy010 29360961

[35] Treangen TJ, Rocha EPC. Horizontal transfer, not duplication, drives the expansion of protein families in prokaryotes. PLoS genetics. 2011; 7: e1001284. DOI: 10.1371/journal.pgen.1001284 21298028 PMC3029252

[36] Stecher B, et al. Gut inflammation can boost horizontal gene transfer between pathogenic and commensal Enterobacteriaceae. Proceedings of the National Academy of Sciences. 2012; 109: 1269–1274. DOI: 10.1073/pnas.1113246109 PMC3268327 22232693

[37] Nathaniel BR, Ghai M, Druce M, Maharaj I, Olaniran AO. Development of a loop-mediated isothermal amplification assay targeting lmo0753 gene for detection of Listeria monocytogenes in wastewater. Letters in applied microbiology. 2019; 69: 264–270. DOI: 10.1111/lam.13200 31323126

[38] Amoah ID, et al. RT-LAMP: a cheaper, simpler and faster alternative for the detection of SARS-CoV-2 in wastewater. Food and environmental virology. 2021; 13: 447–456. DOI: 10.1007/s12560-021-09489-7 34308531 PMC8310731

[39] Ravindran VB, et al. Detection of helminth ova in wastewater using recombinase polymerase amplification coupled to lateral flow strips. Water. 2020; 12: 691. DOI: 10.3390/w12030691

[40] Göpfert L, Elsner M, Seidel M. Isothermal haRPA detection of bla CTX-M in bacterial isolates from water samples and comparison with qPCR. Analytical Methods. 2021; 13: 552–557. DOI: 10.1039/D0AY02000A 33410433

[41] Lupolova N, Dallman TJ, Holden NJ, Gally DL. Patchy promiscuity: machine learning applied to predict the host specificity of Salmonella enterica and Escherichia coli. Microb Genom. 2017; 3: e000135. DOI: 10.1099/mgen.0.000135 29177093 PMC5695212

[42] Lupolova N, Dallman TJ, Matthews L, Bono JL, Gally DL. Support vector machine applied to predict the zoonotic potential of E. coli O157 cattle isolates. Proceedings of the National Academy of Sciences. 2016; 113: 11312–11317. DOI: 10.1073/pnas.1606567113 PMC5056084 27647883

[43] Cook PW, et al. Detection and characterization of swine origin influenza A(H1N1) pandemic 2009 viruses in humans following zoonotic transmission. J Virol. 2020; 95. DOI: 10.1128/JVI.01066-20 PMC7944445 33115872

[44] Cooper N, Griffin R, Franz M, Omotayo M, Nunn CL. Phylogenetic host specificity and understanding parasite sharing in primates. Ecology letters. 2012; 15: 1370–1377. DOI: 10.1111/j.1461-0248.2012.01858.x 22913776

[45] Luis AD, et al. Network analysis of host–virus communities in bats and rodents reveals determinants of cross-species transmission. Ecology letters. 2015; 18: 1153–1162. DOI: 10.1111/ele.12491 26299267 PMC5014217

[46] Wardeh M, Sharkey KJ, Baylis M. Integration of shared-pathogen networks and machine learning reveals the key aspects of zoonoses and predicts mammalian reservoirs. Proc Biol Sci. 2020; 287: 20192882. DOI: 10.1098/rspb.2019.2882 32019444 PMC7031665

[47] Restif O, et al. Model-guided fieldwork: practical guidelines for multidisciplinary research on wildlife ecological and epidemiological dynamics. Ecology letters. 2012; 15: 1083–1094. DOI: 10.1111/j.1461-0248.2012.01836.x 22809422 PMC3466409

[48] Maruyama M, Trung LV. The nature of informal food bazaars: Empirical results for Urban Hanoi, Vietnam. J Retail Consum Serv. 2010; 17: 1–9. DOI: 10.1016/j.jretconser.2009.08.006

[49] Naguib MM, et al. Live and wet markets: food access versus the risk of disease emergence. Trends Microbiol. 2021; 29: 573–581. DOI: 10.1016/j.tim.2021.02.007 33712334 PMC9189808

[50] Kogan NE, et al. Wet markets and food safety: TripAdvisor for improved global digital surveillance. Jmir Public Hlth Sur. 2019; 5: 286–291. DOI: 10.2196/11477 PMC6462893 30932867

[51] Petrikova I, Cole J, Farlow A. COVID-19, wet markets, and planetary health. Lancet Planet Health. 2020; 4: E213–E214. DOI: 10.1016/S2542-5196(20)30122-4 32559435 PMC7832206

[52] Zimmerer KS, de Haan S. Informal food chains and agrobiodiversity need strengthening-not weakening-to address food security amidst the COVID-19 crisis in South America. Food Secur. 2020; 12: 891–894. DOI: 10.1007/s12571-020-01088-x 32837653 PMC7363164

[53] Griggs D, et al. Sustainable development goals for people and planet. Nature. 2013; 495: 305–307. DOI: 10.1038/495305a 23518546

[54] Yuan J, et al. Effect of live poultry market closure on avian influenza A(H7N9) virus activity in Guangzhou, China, 2014. Emerging Infectious Diseases. 2015; 21: 1784–1793. DOI: 10.3201/eid2110.150623 26402310 PMC4593444

[55] Yu HJ, et al. Effect of closure of live poultry markets on poultry-to-person transmission of avian influenza A H7N9 virus: an ecological study. Lancet. 2014; 383: 541–548. DOI: 10.1016/S0140-6736(13)61904-2 24183056 PMC3946250

[56] Lin B, Dietrich ML, Senior RA, Wilcove DS. A better classification of wet markets is key to safeguarding human health and biodiversity. Lancet Planet Health. 2021; 5: E386–E394. DOI: 10.1016/S2542-5196(21)00112-1 34119013 PMC8578676

[57] Li Y, et al. Closure of live bird markets leads to the spread of H7N9 influenza in China. Plos One. 2018; 13. DOI: 10.1371/journal.pone.0208884 PMC6291110 30540847

[58] Nguyen TTT, et al. A stakeholder survey on live bird market closures policy for controlling highly pathogenic avian influenza in Vietnam. Frontiers in Veterinary Science. 2017; 4. DOI: 10.3389/fvets.2017.00136 28879203 PMC5572285

[59] Nadimpalli ML, Pickering AJ. A call for global monitoring of WASH in wet markets. Lancet Planet Health. 2020; 4: E439–E440. DOI: 10.1016/S2542-5196(20)30204-7 33038315 PMC7541042

[60] Zhou XY, et al. The role of live poultry movement and live bird market biosecurity in the epidemiology of influenza A (H7N9): A cross-sectional observational study in four eastern China provinces. J Infection. 2015; 71: 470–479. DOI: 10.1016/j.jinf.2015.06.012 26149187

[61] Chakma S, et al. Risk areas for influenza A(H5) environmental contamination in live bird markets, Dhaka, Bangladesh. Emerging Infectious Diseases. 2021; 27: 2399–2408. DOI: 10.3201/eid2709.204447 34424170 PMC8386803

[62] Guinat C, et al. Optimizing the early detection of low pathogenic avian influenza H7N9 virus in live bird markets. Journal of the Royal Society Interface. 2021; 18. DOI: 10.1098/rsif.2021.0074 PMC8097223 33947269

[63] Paul MC, et al. Quantitative assessment of a spatial multicriteria model for highly pathogenic avian influenza H5N1 in Thailand, and application in Cambodia. Scientific Reports. 2016; 6. DOI: 10.1038/srep31096 27489997 PMC4977984

[64] Mariner JC, et al. Experiences in participatory surveillance and community-based reporting systems for H5N1 highly pathogenic avian influenza: A case study approach. Ecohealth. 2014; 11: 22–35. DOI: 10.1007/s10393-014-0916-0 24643858 PMC4046079

[65] Barnett T, Fournie G. Zoonoses and wet markets: beyond technical interventions. Lancet Planet Health. 2021; 5: E2–E3. DOI: 10.1016/S2542-5196(20)30294-1 33421407 PMC7789916

